# X-Linked Dilated Cardiomyopathy: A Cardiospecific Phenotype of Dystrophinopathy

**DOI:** 10.3390/ph8020303

**Published:** 2015-06-09

**Authors:** Akinori Nakamura

**Affiliations:** 1 Intractable Disease Care Center, Shinshu University Hospital, 3-1-1 Asahi, Matsumoto 390-8621, Japan; E-Mail: anakamu@shinshu-u.ac.jp; Tel.: +81-263-372-673; Fax: +81-263-373-427; 2 Department of Neurology and Rheumatology, Shinshu University School of Medicine, 3-1-1 Asahi, Matsumoto 390-8621, Japan

**Keywords:** dystrophin, *DMD* gene, dilated cardiomyopathy, XLDCM

## Abstract

X-linked dilated cardiomyopathy (XLDCM) is a distinct phenotype of dystrophinopathy characterized by preferential cardiac involvement without any overt skeletal myopathy. XLDCM is caused by mutations of the Duchenne muscular dystrophy (*DMD*) gene and results in lethal heart failure in individuals between 10 and 20 years. Patients with Becker muscular dystrophy, an allelic disorder, have a milder phenotype of skeletal muscle involvement compared to Duchenne muscular dystrophy (DMD) and sometimes present with dilated cardiomyopathy. The precise relationship between mutations in the *DMD* gene and cardiomyopathy remain unclear. However, some hypothetical mechanisms are being considered to be associated with the presence of some several dystrophin isoforms, certain reported mutations, and an unknown dystrophin-related pathophysiological mechanism. Recent therapy for Duchenne muscular dystrophy, the severe dystrophinopathy phenotype, appears promising, but the presence of XLDCM highlights the importance of focusing on cardiomyopathy while elucidating the pathomechanism and developing treatment.

## 1. Introduction

Dystrophinopathy is an X-linked disorder caused by mutations in the *DMD* gene encoding for the sarcolemmal protein dystrophin. Duchenne muscular dystrophy (DMD) and Becker muscular dystrophy (BMD) are representative examples of this disease. The incidence of DMD and BMD is 1/3600 to 6000 and 1/18,000 male births, respectively [1,2]. Patients with the severe form, DMD, present with difficulty in standing and walking abnormality during childhood. Ambulation is affected at approximately 13 years old, and patients finally die of respiratory or cardiac failure. In recent years, the development of mechanical respirators has extended the life expectancy of afflicted patients beyond 30 years of age [1]. Therefore, cardiac involvement has increasingly become a critical issue. Although BMD shows a milder phenotype of skeletal muscle involvement than DMD and patients can generally walk beyond 16 years of age, its severity or course varies among patients.

**Figure 1:**
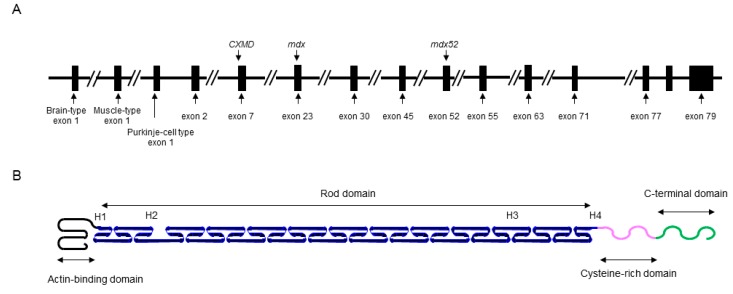
Schematic illustrations of the *DMD* gene and molecular structure of dystrophin protein. (**A**) Key exons in the *DMD* gene are indicated. Mutations of representative DMD model animals are also presented: dystrophic mouse (*mdx*) harboring a nonsense mutation in exon 23, canine X-kinked muscular dystrophy (*CXMD*) with a spice site mutation in intron 7, and exon 52 knock-out mouse (*mdx52*). (**B**) Dystrophin is rod shape structure and is consisted of N-terminal actin-binding domain, rod domain composed of 24 spectrin-like repeats with 4 hinges (H1-H4), cysteine-rich domain, and C-terminal domain.

**Figure 2:**
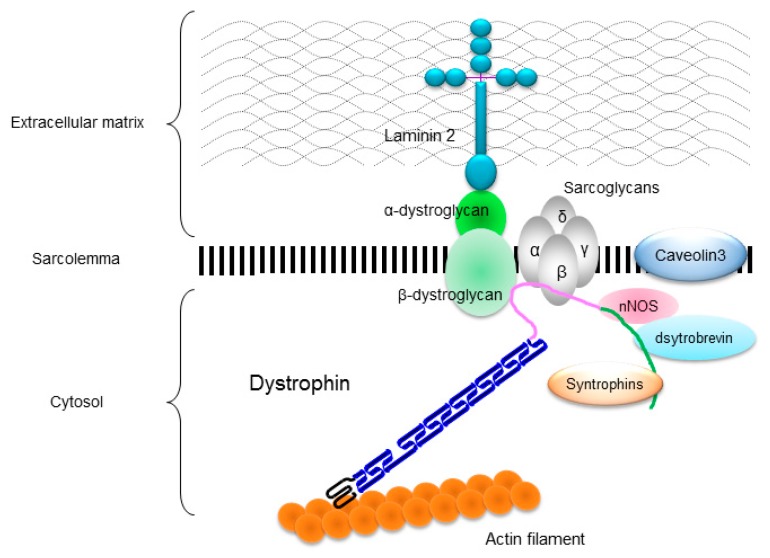
Localization of dystrophin and dystrophin-glycoprotein complex (DGC) in a skeletal muscle cell. Dystrophin links to actin filament at actin-binding domain, and interacts to dystroglycans and sarcoglycan complexes at cysteine-rich domain and to syntrophins, dystrobrevin, and neuronal nitric oxide synthase (nNOS) at C-terminal domain.

The *DMD* gene is located on the human chromosome Xp21 with 79 exons spanning more than 2500 kb (Figure 1A) [3]. Full-length dystrophin is mainly expressed in skeletal, cardiac, and smooth muscles, and the brain. The gene encodes the 427 kDa cytoskeletal protein dystrophin which is a rod-shaped structure consisting of four domains including an N-terminal actin-binding domain (Figure 1B). The rod domain is composed of 24 spectrin-like repeats with four hinges (H1-H4) while a cysteine-rich domain interacts with dystroglycan and sarcoglycan complexes. The C-terminal domain interacts with the syntrophin complex and dystrobrevin. Localized to the sarcolemma, dystrophin is a major component of the dystrophin glycoprotein complex (DGC) along with the dystroglycan, sarcoglycan, and syntrophin/dystrobrevin complexes that link the cytoskeletal protein actin to the basal lamina of muscle fibers (Figure 2) [3,4,5].

DGC is believed to function as a membrane stabilizer during muscle contraction or a transducer of signals from the extracellular matrix to the muscle cytoplasm via its interactions with intracellular signaling molecules [4,5]. Dystrophin deficiency disrupts the DGC and causes the muscle membrane fragility and an increase in susceptibility to the mechanical stress, which leads to progressive muscle necrosis and degeneration in both skeletal and cardiac muscles [6]. However, the underlying mechanism remains unclear.

Patients with dystrophinopathy have various types of mutations such as missense, nonsense, deletion, insertion, or duplication of the *DMD* gene cite as [7]. In cases where an out-of-frame mutation interrupts the reading frame of the coding region and there is no protein production, resulting in the DMD phenotype. If the reading-frame is maintained despite the presence of a mutation (in-frame), a truncated but functional dystrophin is expressed, leading to the BMD phenotype [8]. In the *DMD* gene, there are two hot spots for mutations around exons 3–7 and 45–55 [8,9]. Among patients with dystrophinopathy, some cases present primarily with cardiac manifestations with mild or slight skeletal muscle involvement [10,11,12,13,14,15,16]. X-linked dilated cardiomyopathy (XLDCM), a distinct cardio-specific phenotype of dystrophinopathy, is described in the next paragraph.

## 2. Identification of XLDCM

A distinct dystrophinopathy (OMIM 302045) phenotype, XLDCM presents with congestive heart failure due to dilated cardiomyopathy individuals aged 10–20 years. However, limb and truncal skeletal muscle atrophy and weakness are not always observed. Berko and Swift first reported XLDCM in 1987, within a large family spanning five generations, with 63 male patients diagnosed with DCM [17]. The male patients did not show any skeletal muscle involvement, but showed rapidly progressive heart failure and ventricular arrhythmias between 10 and 20 years old. Further, some male patients showed high serum creatine kinase (CK) levels and some female carriers aged 40–50 years presented with slowly progressing DCM. However, the analysis results of the *DMD* gene and dystrophin expression were not reported in this family.

In 1993, a linkage analysis performed by Towbin *et al.* revealed that the causative gene identified in this family along with the *DMD* gene recognized in another XLDCM family were linked to Xp21.2. Dystrophin expression was dramatically decreased in the patient’s cardiac muscle [18]. In the same year, Muntoni *et al.* reported an XLDCM family having a deletion mutation spanning from a muscle promoter to a portion of intron 1 in the *DMD* gene. These studies established XLDCM as a distinct type of dystrophinopathy [19].

**Figure 3:**
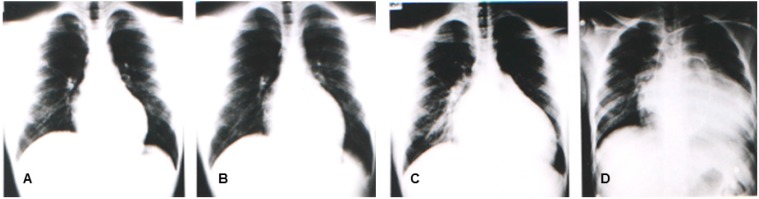
Chest radiographs of a patient having mild BMD with DCM and a l1 insertion mutation in the muscle exon 1 of the *DMD* gene at 12 years (**A**), 15 years (**B**), 17 years (**C**), and 18 years (**D**). Swan ganz catheter is seen on the chest radiograph (**D**).

In the same year, Yoshida, *et al.* reported four patients (from two unrelated families) with congestive heart failure due to DCM who were diagnosed between 10 and 20 years old. The muscle biopsy revealed calf pseudo-hypertrophy, hyper-CKemia, and mild myogenic change [11]. Southern blot and genomic polymerase chain reaction (PCR) analyses revealed that the causative mutation would include a deletion in the region and in exon 1 [11]. Figure 1 illustrates the representative sequential changes in the chest radiographs of one of these patients. Briefly, this patient complained of exertional myalgia of the thighs at 5 years old. At 12 years of age, the patient presented with an elevated CK level of 2510 IU/L (normal range: 62–287 IU/L) without associated muscle weakness. Although muscle atrophy and weakness were not observed throughout the clinical course, the patient complained of exertional dyspnea at 17 years and subsequently died of congestive heart failure at 18 years. The cardio-thoracic ratios in his chest radiographs were at 47%, 50%, 64%, and 71%, at 12, 15, 17, and 18 years of age, respectively (Figure 3). In 1997, Yoshida *et al.* identified an L1 sequence insertion within the muscle type exon 1 in the *DMD* gene of affected members [20]. XLDCM with *DMD* gene mutations or mild BMD with DCM are considered to belong in the same clinical spectrum because of its clinical and phenotypic similarity to XLDCM.

## 3. *DMD* Mutations in Patients with XLDCM

Table 1 summarizes the main reported mutations of the *DMD* gene in patients diagnosed as XLDCM or mild BMD with DCM [9,13,16,19,20,21,22,23,24,25,26,27,28,29,30,31,32,33,34,35]. In Italy, Japan, and the United States, male patients with DCM are being screened for *DMD* gene mutations. In Italy, Arbustini, *et al.* reported that among 201 male patients with DCM, 13 patients (6.5%) had the *DMD* gene mutations within exon 45−55 [32]. Furthermore, 11 patients had increased serum CK levels, and four patients had a family history of DCM. In Japan, Shimizu, *et al.* reported that among 99 male patients with DCM, three patients (3%) had the *DMD* gene mutations [34]. Two patients had an exon 45−48 deletion and one had an exon 48−52 deletion. There were no reported cases of muscle weakness, but serum CK levels ranged from normal to high. Three patients had a family history of sudden death due to DCM or at a young age. The deletion of exon 48−52 is an out-of-frame mutation and predicted to result in the DMD phenotype. Because this study was based on the results of the multiplex-PCR method, the neighboring exon(s) around the deletion may need identification. Feng, *et al.* from the United States reported that among 22 male DCM patients aged 4 to 18 years, three patients (13.6%) had missense mutations [22]. Each patient had a nucleotide base insertion in intron 5 (IVS5+1), a missense mutation (K18N) in exon 2, and a missense mutation (F3228L) in exon 67 [22]. According to Beroud, *et al.* [33] and Nakamura, *et al.* [16], patients with deletions of exons 45−55 covering a whole region that served as a hotspot of mutations, were either asymptomatic or presented with very mild skeletal muscle involvement. Although some cases definitely showed DCM or congestive heart failure [16,33], the severity of the DCM was relatively mild and patients responded well to a β-blocker or angiotensin-converting enzyme inhibitors (ACE-I). Recently, two male siblings with BMD developed severe DCM at 11 years old and one of them died at 14 years old. These patients had a novel frameshift mutation in exon 27 of the *DMD* gene (c.3779_3785delCTTTGGAinsGG). Although this predicts an amino-acid substitution and premature termination (p.Thr1260Argfs*8), muscle biopsy dystrophin immunostaining indicated that the mutation is more likely to alter splicing [29].

## 4. Pathogenic Mechanisms in XLDCM

Based on the previous case reports, the *DMD* gene mutations occurring with XLDCM or mild BMD with DCM, have been classified into the following four regions: (1) The region from the muscle promoter to muscle exon 1; (2) The region from exon 2−8 region coding the actin binding domain; (3) The region from exon 45−55, (considered the “hot spot” for mutations in the *DMD* gene) coding the rod domain; and (4) The remaining region. The following proposed hypotheses associated with the *DMD* gene mutations potentially explain the possible pathogenesis of cardiospecific involvement.

### 4.1. Hypothesis 1: The Transcriptional Regulation of the DMD Gene Differs between the Skeletal and Cardiac Muscles

Currently, three different isoforms of the full-length dystrophin are expressed in the skeletal and cardiac muscle (muscle isoform), in the central nervous system (brain isoform), and in the cerebellar Purkinje-cells (Purkinje-cell isoform).

**Table 1:** X-linked dilated cardiomyopathy and dilated cardiomyopathy with mild Becker muscular dystrophy having the *DMD* mutations.

.	Mutation type	Family history	Serum CK level	Reference [No.]
Muscle promoter-exon 1	Deletion	+	High	Muntoni, *et al.* 1993 [19]
Muscle exon 1	Insertion of L1sequence	+	High	Yoshida, *et al.* 1993 [11], 1998 [20]
Intron 1	Point mutation at splice donor site	+	Normal~High	Milasin, *et al.* 1996 [21]
Exon 2	Missense mutation	−	High	Feng, *et al.* 2002 [22]
Exon 2-7	Deletion		High	Gold, *et al.* 1992 [23), Nigro, *et al.* 1995 [24]
Exon 2-7	Duplication	+	High	Bies, *et al.* 1997 [25]
Exon 3-7	Deletion		Unknown	Nigro, *et al.* 1995 [24]
Intron 5	IVS5+1	−	Unknown	Feng, *et al.* 2002 [22]
Exon (5) 6-13(14/15)	Deletion (actual range was unknown)	−	Unknown	Oldfors, *et al.* 1994 [26]
Exon 9	Missense mutation	+	High	Ortiz-Lopez, *et al.* 1997 [27]
Intron 11	Insertion of *Alu* like sequence	+	High	Ferlini, *et al.* 1998 [28]
Exon 27	Frameshift mutation	+	High	Tsuda, *et al.* 2014 [29]
Exon 27-30	Deletion		High	Franz, *et al.* 2000 [30]
Exon 29	Missense mutation	+	High	Franz, *et al.* 2000 [31]
Exon 45-51	Deletion	+	High	Arbustini, *et al.* 2000 [32]
Exon 45-55	Deletion	+/−	Normal~High	Beroud, *et al.* 2007 [33]; Nakamura, *et al.* 2008 [16]
Exon 45-48	Deletion	−	High	Arbustini, *et al.* 2000 [32]; Shimizu, *et al.* 2005 [34]
Exon 48	Deletion	−	Normal	Arbustini, *et al.* 2000 [32]
Exon 48-51	Deletion	−	High	Arbustini, *et al.* 2000 [32]
Exon 48-49	Deletion	+/−	High	Piccolo, *et al.* 1994 [13], Muntoni, *et al.* 1997 [35]
Exon 48-52	Deletion	+	Normal	Shimizu, *et al.* [34]
Exon 48-53	Deletion	−	Normal	Arbustini, *et al.* 2000 [31]
Exon 49-51	Deletion	−	Normal	Gold, *et al.* 1992 [23], Muntoni, *et al.* 1997 [35]
Exon 67	Missense mutation	−	Unknown	Feng, *et al.* 2002 [22]

According to Muntoni, *et al*, a deletion from the muscle promoter to a part of intron 1 in XLDCM, resulted in a missing muscle-isoform with overexpression of the brain and Purkinje-cell isoforms but not in the cardiac muscle [36,37]. In the skeletal muscle of two families with L1 insertion mutations in the muscle exon 1 reported by Yoshida, *et al.* [11,20], the absence of the muscle isoform expression resulted in the compensatory overexpression of the brain and Purkinje-cell isoforms [38]. Thus, the transcriptional regulation of dystrophin isoforms differs between skeletal and cardiac muscles resulting in phenotypic differences. A recent report by Neri, *et al.* stated that the muscle isoform is expressed in normal ventricular muscles, while brain isoform is expressed in both the atria and conduction system but not in the ventricular muscles. The ventricular dilatation seen in XLDCM with a mutation in the 5’ end of the *DMD* gene appears to be functionally related to loss of the muscle isoform, the only isoform transcribed in human ventricles; in contrast, the brain isoform is well expressed in heart but confined to the atria. Since the brain isoform can functionally replace the muscle isoform in the skeletal muscle, its expression in the heart could potentially exert the same rescue function [39].

On the other hand, Ferlini, *et al.* has reported an XLDCM family with an insertion mutation of *Alu*-like sequences in intron 11 [28]. The skeletal muscle of the affected members included both the normal and mutated dystrophin mRNA but only the variant dystrophin mRNA caused by splicing was expressed in the cardiac muscle. Thus, the distinct phenotype might be due to the differences in the transcriptional regulation of dystrophin mRNA between the skeletal and cardiac muscles.

### 4.2. Hypothesis 2: Dystrophin Stability and Protein-Binding Mechanisms Differ between the Skeletal and Cardiac Muscles

The cardiospecific pathogenesis for the “hot spot” mutations in exon 45−55 in dystrophinopathy remains unknown. Since exon 48 is a notable exception in these cases, any cis-regulatory elements for cardiac muscles are believed to exist in intron 48 [35,40]. The rod-domain 16/17 (R16/17) coding by exon 42−44 has another biding site for neuronal nitric oxide synthase (nNOS), which connects with the C-terminal domain of dystrophin [41]. Although the effect of nNOS on selective cardiac damage has not been elucidated, it is interesting to see the differences in the role of nNOS between skeletal and cardiac muscles.

A certain mutation may change the conformational cardiospecific stability of dystrophin or the interaction between dystrophin and its binding proteins. An XLDCM family reported by Ortz-Lopez, *et al.* had an amino acid replacement in the hinge 1 (H1) region of dystrophin. He proposed that the H1 region might play an important role in cardiac function and structure [27]. Franz, *et al.* reported an XLDCM family with missense mutation in exon 29. The family showed a decrease in dystrophin up to 20% of the normal levels in both the skeletal and cardiac muscles while the β-and δ-sarcoglycans were distinctly decreased in the cardiac muscle [30]. Therefore, structural changes in dystrophin may result in a deteriorated relationship between dystrophin and dystrophin-related proteins.

Bies, *et al.* reported a lack of dystrophin and α-dystroglycan in the myocardial cell membrane fractions of a patient with XLDCM and a duplication of the exon 2−7 mutation [25]. Missense mutations (F3228L) in exon 67 were reported by Feng in the dystroglycan binding region [22] resulting in different dystroglycan expressions between the skeletal and cardiac muscles. According to Singh, *et al.*, the missense mutation (K18N) in exon 2, which was initially reported by Feng, *et al.* [22] may not affect the functionality of dystrophin but instead, decrease its stability by increasing anomalous folding and structural instability [42]. From the analyses of XLDCM cases, the structure of dystrophin and its stability may relate to the pathogenesis of cardiac involvement.

### 4.3. Hypothesis 3: Exercise Overload Affects Cardiac Damage

Towbin *et al* have proposed the so-called “use phenomenon” that states that myocardial damage is induced by exercise. [43]. It is a hypothesis that repeated cardiac muscle contraction and relaxation occurring during slight skeletal muscle movement and physical exercise further increases cardiac overload resulting in progression of cardiac damage. Melacini, *et al.* reported myocardial involvement in many cases of subclinical BMD. Unaware of a possible cardiac involvement, patients continue to perform strenuous muscle exercises and pressure or volume overload induces mechanical stress on the dystrophin-deficient myocardium [15]. In an experimental model, Nakamura, *et al.* reported that physical exercise progresses myocardial damage in dystrophin-deficient *mdx* mice [44].

## 5. Clinical and Laboratory Features in XLDCM

As described above, the age of onset of dilated cardiomyopathy with congestive heart failure is between 10 to 20 years old in XLDCM. Before cardiac symptoms become apparent, abnormal findings in the electrocardiogram, chest radiograph, or high-CKemia are often noticed during screening. XLDCM manifests as cramping myalgia on physical exercise or calf hypertrophy, but muscle weakness and atrophy of the limbs or trunk are not observed. In many cases, serum CK level is slightly or mildly elevated suggesting subclinical muscle damage, but may be within normal levels in some cases. The muscle biopsy revealed slight myogenic changes including round atrophy, varied fiber size, necrosis, and centronuclear fibers [16,26].

On the other hand, cardiac involvement substantially progresses and many patients with so-called “XLDCM” die of heart failure between 10 and 20 years old. The first case of a 50-year-old diagnosed with XLDCM and normal serum CK levels has also been reported [16,33]. Such cases have mutations in the “hot-spot” region of the *DMD* gene, and they usually have a good life expectancy.

Myocardial biopsy of the dystrophin-deficient cardiac muscle shows the replacement of muscle with fibrotic or fatty tissue, especially in the left ventricular posterobasal wall region [45,46,47,48,49]. Atrophic changes with loss of striation, vacuolation, fragmentation, or nuclear degeneration in the myocardium have also been reported [50]. Progressive involvement of the left ventricle leads to wall motion abnormality and results in dilated cardiomyopathy.

The electrocardiogram (ECG) in dystrophinopathy shows high R waves (R/S > 1) in the right precordial leads (V_1-2_), deep Q waves in leads I, aV_L_, and V_5-6_ or in leads II, III, and aV_F_ [45,46,47,48,49,50,51]. An increased heart rate, shortened PQ (PR) interval, conduction abnormalities or arrhythmias such as sinus arrhythmia, atrial ectopic beats, and ventricular premature complexes are observed in the ECG of patients with dystrophinopathy [51,52,53,54]. Echocardiography results include myocardial thickening, wall motion abnormalities, enlargement of the left ventricle, and left ventricular systolic or diastolic dysfunction [52,53].

## 6. Diagnosis and Differential Diagnosis

When DCM is observed in young male patients, it is quite important to ask for a family history of the disease while considering the diagnosis of XLDCM. Middle-aged female patients are often included in the family assessment, and it is important to highlight the differences in the age and severity of symptoms between male and female patients. Because 1/3 of patients with dystrophinopathy do not a have family history of the disease, some patients may not be aware of the diagnosis. Measurement of the serum CK level may help with the diagnosis.

The definitive diagnosis is defined by the identification of the *DMD* gene mutations by gene analysis such as multiplex-PCR or multiplex ligation dependent probe amplification (MLPA). Although mutation detection rate by MLPA is higher than Southern blotting or multiplex-PCR [55,56], it is not always able to detect micro-deletions or point mutations. Although this method is expensive, sequencing techniques involving all *DMD* exons including those at the intro-exon boundaries, will help identify the mutation in these cases. Muscle or right ventricular myocardial biopsy is available for confirming the lack of or reduction in dystrophin, but either method is invasive and involves many life-threatening risks.

Differential diagnosis includes various disorders presenting as DCM. Because 25 to 30% of DCM is considered as hereditary, some of the representative syndromic conditions with associated DCM were summarized in Table 2. Among these, Barth syndrome, McLoad syndrome, and Danon disease (glycogen storage disease type IIb) are X-linked disorders. Other syndromic or non-syndromic hereditary forms with known disease gene are referred to a review reported by Hermans, *et al.* [57], and the GeneTable of Neuromuscular Disorders [58] disease group 10.

Differential diagnoses for Acquired DCM include raised coronary artery disease, myocardial infarction, valvular disease, congenital heart disease, toxins (such as anthracycline), thyroid disorders, inflammatory conditions, myocarditis, severe hypertension, *etc.* The differential diagnoses of acquired DCM is determined by taking the history, physical examination, and laboratory data such as blood examination, ECG, echocardiography, or coronary angiography. Protease 2A released by the coxsackievirus can cleave dystrophin resulting in myocarditis, illustrating one of the common pathogeneses of myocarditis [59]. Additionally, it has been reported that dystrophin deficiency markedly increases enterovirus-induced cardiomyopathy [60] such that the viral infection can influence the severity and penetrance of the cardiomyopathy that occurs in patients with dystrophinopathy.

## 7. Treatment

The treatment for cardiac involvement of XLDCM is similar to that for DCM. The first-line drugs for the management of chronic heart failure are angiotensin converting enzyme inhibitor (ACE-I) and β-blockers, with improved prognosis in DMD patients. [61,62,63]. Angiotensin II contributes to the fibrogenesis by enhancing a fibrogenetic cytokine tumor growth factor (TGF)-βI in heart [64]; therefore, ACE-I has been more extensively used. While, β-blockers have also been frequently used in patients with DCM, and the combination therapy of ACE-I and β-blocker provided a significant improvement on left ventricular fractional shortening compare to ACE-I alone [65]. The reduction of the after load is also believed to have guided the improvement in cardiac function [61,62,63].

**Table 2:** Differential diagnosis of X-linked dilated cardiomyopathy.

Disease	Inheritance	Gene	Symptoms and laboratory examination
Emery-Dreifuss muscular dystrophy	AR or AD	*LMNA*	joint contracture, hyper-CKemia, arrhythmias, muscle weakness of childhood onset
LGMD 1B	AR	*LMNA*	joint contracture (mild), hyper-CKemia, arrhythmias, weakness of limb-girdle muscle
McLoad syndrome	X-linked	*XK*	chorea, myopathy, hyper-CKemia, ancanthocyte
Barth syndrome	X-linked	*TAZ*	growth retardation, lactic acidosis, leukocytopenia, increase in 3-methylglutacon levels
Danon disease	X-linked	*LAMP-2*	limb muscle weakness, atrophy and myalgia, hypertrophic or dilated cardiomyopathy, arrhythmias, mental retardation
Laing dystal myopathy	AD	*MYH7*	childhood onset, Involvement of face, ankle, thumb, digital extensor, neck flexor muscles
Carvajal syndrome	AR	*DSP*	palmoplantar keratosis, kinky hair
Mitochondrial dilated cardiomyopathy	Maternal inheritance	*mtDNA*	focal glomerulosclerosis, Kearns-Sayre syndrome
HFE gene-related hereditary hemochromatosis	AD	*HFE*	liver cirrhosis, debates mellitus, deposition of melanin, increase in serum Fe and ferritin, DCM due to siderosis

In contrast, the efficacy of angiotensin II receptor blockers (ARBs) for DCM with dystrophinopathy has not been confirmed; however, it has been suggested that ARBs have therapeutic effect in animal model of DCM [66,67]. Diuretics and digoxin are most effective when congestive heart failure becomes apparent. The use of corticosteroids in DMD can be useful not only for the skeletal but also cardiac muscles [68,69]. However, corticosteroids are not currently recommended for XLDCM patients.

Surgical treatment may be necessary in cases of intractable heart failure. Treatment with an artificial pacemaker, cardiac resynchronization therapy (biventricular pacing), is appropriate for cases of heart failure with associated ventricular dyssynchrony [70]. Partial left ventriculectomy (Batista procedure) [71] and left ventricular assist devices [72,73], in addition to cardiac transplantation, have been reported for the management of BMD-associated cardiomyopathy [74,75].

Currently, the restoration of dystrophin expression in the skeletal and cardiac muscle using gene transfer by viral vectors is a promising avenue for therapy [76,77]. Recently, truncated dystrophin gene constructs have been engineered and shown to alleviate dystrophic cardiac muscle disease [78,79]. Taghli-Lamallem, *et al.* reported that either the mechanical or the signaling functions of dystrophin reduced the dilated heart phenotype of dystrophin mutants in a drosophila model, and dystrophin retained some function in the absence of a predicted mechanical link to the cytoskeleton [79]. Cardiospecific manipulation of NOS expression modulates cardiac function, which can be reversed in part by the loss of the dystrophin function, further supporting the potential signaling role of dystrophin in the heart [80].

To date, exon-skipping mediated by antisense oligonucleotides (AOs) has been promising therapeutic approach for DMD [81]. The exon-skipping therapy changes an out-of-frame mutation into an in-frame mutation by skipping exon(s) nearby the mutation using AOs, and restores truncated dystrophin expression. Consequently, a severe dystrophic phenotype could convert into a milder phenotype. Recently, stable and less-toxic AOs have been developed, and their efficacies have been confirmed in mice DMD models (*mdx* and *mdx52*) and dog DMD models (canine X-linked muscular dystrophy: *CXMD*) [82,83,84]. The focus of clinical trials has primarily been on evaluating the effect of treatments on the skeletal muscle [85,86]. The half-life of exon-skipping drugs is longer in the heart but the delivery to cardiomyocytes is less efficient than for skeletal muscle cells. This is because the DMD membrane in the skeletal muscle is more leaky than in cardiomyocytes, and allows for better uptake of the skipping drugs [87]. These issues could alter the ability to restore dystrophin expression in the heart. Therefore, access to human cardiomyocytes with various *DMD* mutations would accelerate preclinical development of therapeutics. Recently, human induced pluripotent stem cells (hiPSCs) from skin fibroblasts derived from DMD patients harboring out of frame deletions or nonsense point mutations in the *DMD* gene were induced to differentiate into electrophysiologically and pharmacologically functional cardiomyocytes. Delivery of AOs targeted to exon 51 of the *DMD* gene induced exon skipping in DMD hiPSC-cardiomyocytes, leading to the expression of the dystrophin protein to a level of ∼30% of wild-type cells [88]. Viral transduction of DMD hiPSC-cardiomyocytes led to the expression of a dystrophin minigene at the RNA and protein level up to 90% of normal dystrophin levels.

As a quite new strategy for the management of dystrophin-related cardiomyopathy, Metzger and coworkers presented an *in vivo* administration of chemical-based membrane sealant poloxamer 188 that improved the ventricular geometry in dystrophic mice [89] and chronically prevented cardiac injury and dilatation in dystrophic dogs [90]. This sealant could propose a new therapeutic approach for preventing or reversing the progression of cardiomyopathy and heart failure in dystrophinopathy.

At this moment, there are few *in vitro* experiments for cardiac muscle of patients having XLDCM or mild BMD with DCM because primary cardiomyocytes are not easily available for repeated experiments on identical cultures [88]. In the future, an *in vitro* system for cardiac muscles in parallel with skeletal muscle would be beneficial and important to examine the differences in pathophysiology, therapeutic efficacy, and drug-induced toxicity between them. In this respect, the hiPSC-cardiomyocytes can be useful for evaluating the efficacy of novel therapeutics for DCM due to dystrophinopathy.

## 8. Conclusions

The prognosis of DMD has been prolonged due to the administration of corticosteroids and the introduction of the artificial respirator, and the development of various therapeutic approaches. Cardiac failure and lethal arrhythmias are critical issues that need to be addressed in cases of XLDCM or mild BMD with DCM from the beginning. There is an urgent need for therapy that can rescue dystrophin expression in cardiac muscles. Further studies of cases with XLDCM and mild BMD with DCM will be quite important not only to examine the differences in pathophysiological mechanism between skeletal and cardiac muscles, but also to develop the therapeutic strategies for cardiac involvement.

## Abbreviations

XLDCM: X-linked dilated cardiomyopathy
DCM: dilated cardiomyopathy
DMD: Duchenne muscular dystrophy
BMD: Becker muscular dystrophy
DGC: dystrophin glycoprotein complex
nNOS: neuronal nitric oxide synthase
CK: creatine kinase
PCR: polymerase chain reaction
IVS: intervening sequence
ECG: echocardiography
MLPA: multiple ligation probe amplification
ACE-I: angiotensin converting enzyme inhibitor
TGF: tumor growth factor
ARB: angiotensin receptor II blocker
AOs: antisense oligonucleotides
hiPSCs: human induced pluripotent stem cells
